# Evaluating Temporal Consistency in Marine Biodiversity Hotspots

**DOI:** 10.1371/journal.pone.0133301

**Published:** 2015-07-22

**Authors:** Susan E. Piacenza, Lindsey L. Thurman, Allison K. Barner, Cassandra E. Benkwitt, Kate S. Boersma, Elizabeth B. Cerny-Chipman, Kurt E. Ingeman, Tye L. Kindinger, Amy J. Lindsley, Jake Nelson, Jessica N. Reimer, Jennifer C. Rowe, Chenchen Shen, Kevin A. Thompson, Selina S. Heppell

**Affiliations:** 1 Department of Fisheries and Wildlife, Oregon State University, Corvallis, OR, United States of America; 2 Department of Integrative Biology, Oregon State University, Corvallis, OR, United States of America; 3 Department of Geography, Environmental Sciences and Marine Resource Management, Oregon State University, Corvallis, OR, United States of America; 4 Department of Biology, University of San Diego, San Diego, CA, United States of America; 5 Department of Information Systems, Drexel University, Philadelphia, PA, United States of America; UC Santa Cruz Department of Ecology and Evolutionary Biology, UNITED STATES

## Abstract

With the ongoing crisis of biodiversity loss and limited resources for conservation, the concept of biodiversity hotspots has been useful in determining conservation priority areas. However, there has been limited research into how temporal variability in biodiversity may influence conservation area prioritization. To address this information gap, we present an approach to evaluate the temporal consistency of biodiversity hotspots in large marine ecosystems. Using a large scale, public monitoring dataset collected over an eight year period off the US Pacific Coast, we developed a methodological approach for avoiding biases associated with hotspot delineation. We aggregated benthic fish species data from research trawls and calculated mean hotspot thresholds for fish species richness and Shannon’s diversity indices over the eight year dataset. We used a spatial frequency distribution method to assign hotspot designations to the grid cells annually. We found no areas containing consistently high biodiversity through the entire study period based on the mean thresholds, and no grid cell was designated as a hotspot for greater than 50% of the time-series. To test if our approach was sensitive to sampling effort and the geographic extent of the survey, we followed a similar routine for the northern region of the survey area. Our finding of low consistency in benthic fish biodiversity hotspots over time was upheld, regardless of biodiversity metric used, whether thresholds were calculated per year or across all years, or the spatial extent for which we calculated thresholds and identified hotspots. Our results suggest that static measures of benthic fish biodiversity off the US West Coast are insufficient for identification of hotspots and that long-term data are required to appropriately identify patterns of high temporal variability in biodiversity for these highly mobile taxa. Given that ecological communities are responding to a changing climate and other environmental perturbations, our work highlights the need for scientists and conservation managers to consider both spatial and temporal dynamics when designating biodiversity hotspots.

## Introduction

As global biodiversity loss continues at an unprecedented rate [1], protection of vulnerable species and ecosystems is of paramount concern. In order to prioritize conservation efforts with limited resources, Myers et al. (2000) first proposed the concept of “biodiversity hotspots” to identify areas with exceptional concentrations of endemic species that are also highly threatened–a watershed concept in conservation biology that has influenced many research, conservation and resource management programs [2,3]. Subsequent interpretations of hotspots have more simply defined them as areas of high species diversity [4], particularly in systems where quantitative measures of threat levels are difficult to assess, for systems that are unilaterally threatened, or have few endemic species, such as coral reefs [5,6].

Nested within the hotspot concept is the assumption of stability in biodiversity over time, most likely from its terrestrial application allowing for delineation of tangible boundaries [3]. However, variation in biotic and abiotic factors is known to affect species distributions, richness, and abundance across spatial and temporal scales [4,7], particularly in marine ecosystems (e.g., [8–12]). For example, many ecosystems undergo periodic natural disturbance regimes (i.e. fire, storms, etc.), while others can be characterized by successional stages and/or alternative states [13], resulting in a dynamic exchange of individuals across the landscape both in space and time. Additionally, dispersal and migratory patterns may encompass broad geographic regions, or cross ecological boundaries, with the potential for significant inter- and intra-annual fluctuations in local community structure and function [14–17]. While these patterns are largely observable, anthropogenic drivers of biodiversity change (e.g. habitat alteration, overexploitation, introduction of exotic species, and climate change) fluctuate in both occurrence and intensity through time and are predicted to disrupt this synchrony in nature [18]. In particular, climate models predict increased variability and frequency of climatic extremes over multiple time-scales, from changes that are sudden (occurring in less than five years), to changes in climate over the next century [19]. This variability is expected to drive shifts in species’ distributions and, consequently, the composition of ecological communities [12,20]. For ecosystems that are inherently driven by cyclical or periodic variation in the environment, this continuous change may result in future no-analog conditions [21].

The typical application of biodiversity hotspots via discrete measurements of biodiversity, as opposed to evaluation of candidate areas through time [2,4,13], has resulted in a bias toward candidate hotspot areas exhibiting high biodiversity during the initial assessments. Consequently, this can lead to the designation of biodiversity hotspots that are not reflective of prevailing conditions (i.e. a cyclical or periodic disturbance regime has temporarily inflated or deflated biodiversity levels), or that may show substantial decline in biodiversity with continued anthropogenic change. Furthermore, the biodiversity levels used to identify hotspots are often user-defined or set at arbitrary thresholds that are rarely based on long-term ecological data [22].

To address these issues of temporal disregard and subjectivity in hotspot designation, we present an objective approach for identifying biodiversity hotspots via a case study, wherein the assessment of temporal variability in biodiversity was integrated into the identification of candidate hotspots. We applied this approach to an eight-year dataset of benthic fish populations in offshore soft sediment habitat of the Northeast Pacific Ocean, within the California Current Large Marine Ecosystem (CCLME). The CCLME is an oceanic region of over two million km^2^ that borders the west coast of North America and encompasses both temperate and subtropical climatic zones with strong seasonal and inter-annual variability [23]. Inter-decadal patterns introduce additional variability in oceanic conditions, influenced by large-scale climatic cycles, such as the El Nino Southern Oscillation (ENSO) and the Pacific Decadal Oscillation (PDO) over longer time scales [24,25]. Many vertebrate taxa inhabiting this region have developed adaptations to this strong seasonal variability, including the capacity for long-distance dispersal. Given this high environmental variability and highly vagile fauna, we suspected that the conventional view of hotspots as temporally and spatially static may not apply in this system.

Using this dataset, we determined threshold levels for hotspot candidacy and defined hotspots by their consistency in benthic fish biodiversity through time. We hypothesized that fish biodiversity levels would fluctuate through time across the CCLME. To qualify as a hotspot in this study, a given area must maintain biodiversity levels above the threshold over the duration of the study period. Additionally, we provide a general framework to be used in the temporal evaluation of biodiversity hotspots in other long-term, large-scale datasets.

## Materials and Methods

### General Approach

**Fig 1:**
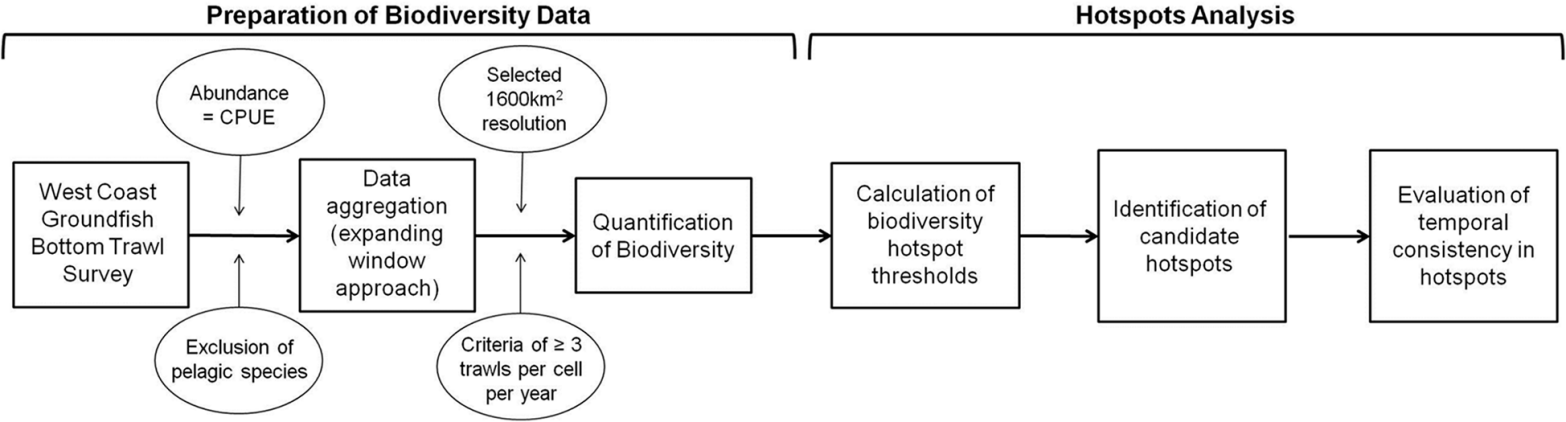
A flowchart of decision points (circled text) and methodological steps (boxed text) used to assess temporal consistency in biodiversity hotspots.

We used a dataset from US federal marine trawl surveys to examine spatial variability in the distribution of fish biodiversity in the CCLME, quantified biodiversity hotspots meeting our threshold criteria annually, and evaluated the potential for temporal consistency in biodiversity throughout our study period. We provide a flowchart to outline the general methodological steps (Fig 1), which can be used as a template for future studies with long-term, large-scale data.

### West Coast Groundfish Bottom Trawl Survey (WCGBTS)

We obtained fish catch records from the 2003–2010 West Coast Groundfish Bottom Trawl Survey (WCGBTS) administered by the Northwest Fishery Science Center, National Oceanic and Atmospheric Administration (NOAA), US [26]. The WCGBTS is a scientific trawl survey conducted each year from mid-May until late October. We tested for seasonal differences, using generalized linear models, and survey month was not a significant explanatory variable (p(richness) = 0.643 and p(H') = 0.127); therefore, we did not consider monthly effects in further analyses. In each trawl survey, crew members use bottom trawl fishing gear, with an Aberdeen-type net, with a small mesh (3.81cm^2^) codend liner, to collect benthic organisms and identify them to *Genus species*. The survey follows a stratified-random sampling design and includes three depth zones (55–183 m, 184–549 m, and 550–1,280 m) from Cape Flattery, WA south to the US-Mexico border along the CCLME. Within each year, the WCGBTS randomized trawl survey locations within each depth stratum based on a 10 km^2^ grid of the entire CCLME. Thus, sampling does not occur in the same locations every year. For this study we used survey data from 2003–2010, with a total of 5,162 surveys. We then standardized species abundances as biomass over area swept (kg/ha) to approximate catch per unit effort (CPUE). We excluded pelagic species from our analysis because the fishing gear was designed to collect benthic species and the pelagic habitat sampled was not standardized by trawl area swept (S1 File).

### Biodiversity Indices

We calculated benthic fish species richness (hereafter richness) and Shannon diversity (H′) for each trawl haul. Species richness was the total number of species present in a sample after removing pelagic species. Shannon diversity was calculated as the sum of proportional biomass/area swept (kg/ha) of all benthic fish species in a sample:

$$H\prime =\sum_{i=1}^{n}p_{i}\;\text{ln}\;p_{i}$$ (1)

where *p*_*i*_ is the proportion of biomass (CPUE) of species *i* in a trawl [24]. We chose this measure for two reasons: our data were measured as biomass, rather than counts of individuals/species, which precluded the use of many biodiversity indices, and H′, in which biomass units have been applied, is a commonly used, readily comparable, and interpretable index of biodiversity [27,28].

### Aggregating Trawl Data

**Fig 2:**
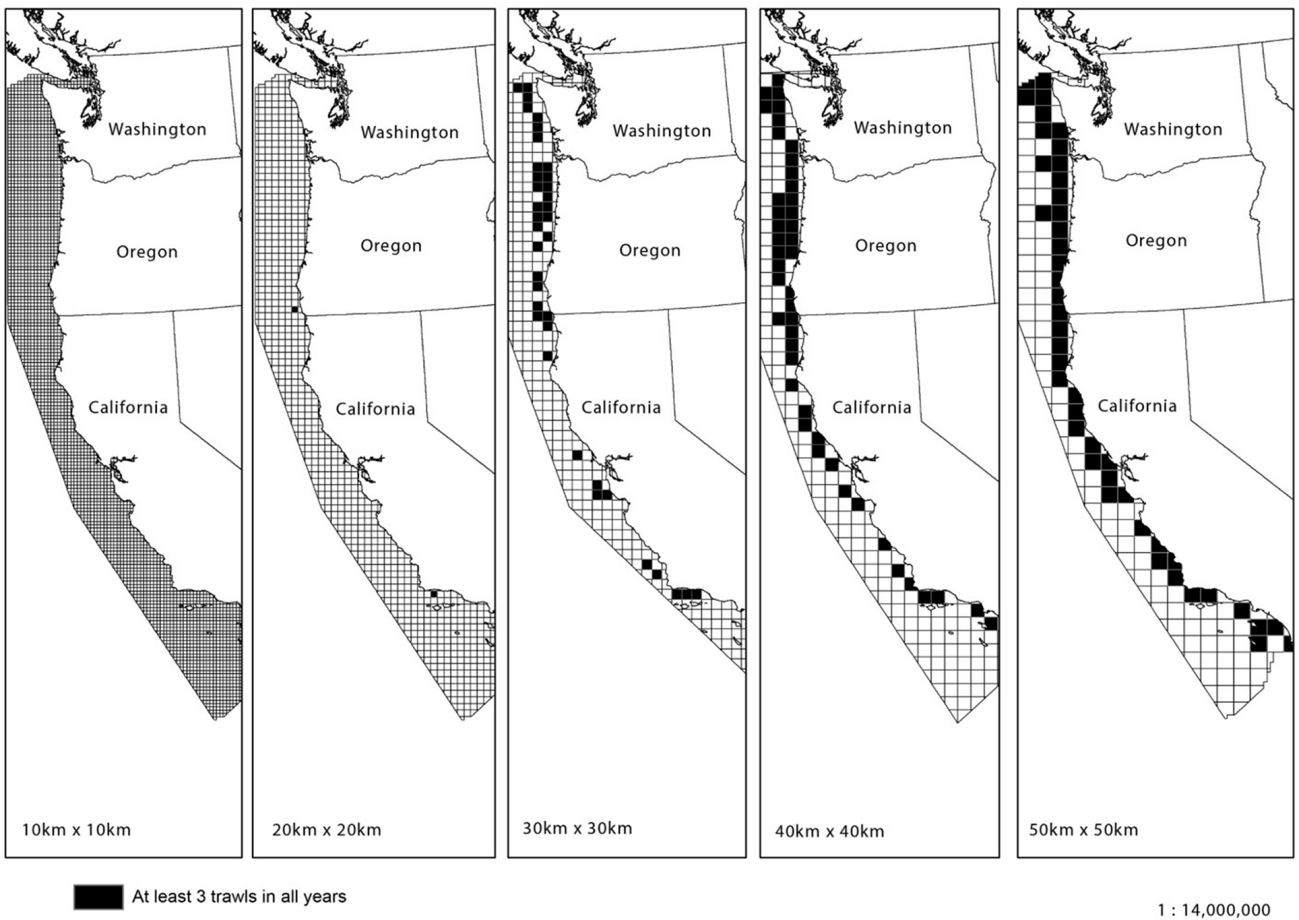
Expanding window approach used to identify cells that contained at least three trawls for each year (2003–2010). 1600 km^2^ (40 x 40 km) cells offered both the highest number of cells that qualified, as well as a high degree of spatial connectivity between the cells.

The number of trawls per 100 km^2^ grid cell varied by year because trawl locations surveyed in each year were randomized. We eliminated cells that contained less than three trawls in any one year and aggregated the trawl data to grid cells of varying sizes using an expanding window method in ArcGIS 10.1, using open-access geospatial data from the US Census Bureau. Our goal was to produce the largest number of grid cells for each year of the survey while still using a scale that maintains biological relevance in our system. We compared grid cells of varying sizes and determined that the 1600 km^2^ grid resolution (40 x 40 km) provided the best compromise between spatial coverage, resolution and biological relevance. This yielded a total of 46 grid cells that could be analyzed for all years (Fig 2).

After aggregating trawl data into grid cells, the number of trawls per grid cell within each year ranged from 3–22. To reduce the inherent bias of uneven sampling effort on biodiversity metrics, we performed a randomized re-sampling procedure using the package *vegan* in R Version 3.0.1. We re-sampled three randomly-drawn trawls, with replacement, 100,000 times within each grid cell and calculated average richness and H′ for each iteration. We repeated this procedure for trawls performed within each grid cell for all eight years combined, as well as within each year. We examined frequency distributions of the randomized iterations of both diversity measures to check for skewness: they were consistently normal across all grid cells, regardless of the number of trawl samples in the cell. This remained the case regardless of whether random resampling was conducted on each year individually or all years combined. We compared the relationship between the number of trawls per grid cell and the mean richness and H′ values for each grid cell when using the raw mean diversity values versus the mean diversity values from the randomly sampled frequency distributions (S1 Fig). We observed a significant positive relationship between sampling effort and the raw diversity values (simple linear regression: richness, slope = 1.4679, p < 0.001; H′, slope = 0.02976, p < 0.001), indicating an effect of sampling effort. This relationship, however, became insignificant after our randomized sampling procedure (simple linear regression: richness, p = 0.80; H′, p = 0.27). Thus, we used these mean biodiversity values from the frequency distributions of randomized sampling to represent biodiversity for each grid cell for hotspot delineation (S2 and S3 Figs). We plotted Species Accumulation Curves (SACs) to determine if there was sufficient sampling for fish over each year [29]. The SACs for all years combined reached asymptotes in all cases, indicating no need to employ rarefaction to accurately estimate true species richness. Also, traditional sample-based rarefaction procedures were inappropriate for our data because of the large number of grid cells with only three trawls per year [30].

#### Defining biodiversity hotspots

We designated hotspots following a spatial frequency distribution method developed by Bartolino et al. (2010) [31], in R Version 3.0.1. This method has an advantage over traditional spatial hotspot delineation techniques in that a hotspot threshold is defined from the geometric properties of a cumulative relative frequency distribution (CRFD) curve rather than a more subjective user-defined threshold [31–34]. For each year (2003–2010), we approximated a CRFD curve by plotting the relative value of the biodiversity measure (species richness or Shannon diversity, H′) against the frequency distribution for that value (S2 File) [31]. We derived a yearly threshold diversity value that corresponded to the point on the curve where the relative increase in biodiversity was equal to the relative increase in the area sampled. This threshold represents a natural boundary between regions with lower and higher biodiversity accumulation rates (S2 File) [31].

#### Evaluation of temporal variability

To examine consistency of hotspots over the entire spatial and temporal extent of the dataset, we used a mean threshold standardized across all years (2003–2010), hereafter, “universal threshold.” This allowed for the evaluation of consistency of hotspots through time, as each grid cell was categorized as “hot” (exceeding the universal threshold) or “not hot”(below the universal threshold) for any given year. We present results based on the *mean* universal threshold (as opposed to the minimum or maximum), although we also examined the number and consistency of hotspots identified using all three universal thresholds. We also compared the hotspots derived from the universal mean threshold method with those derived from yearly thresholds. The mean universal threshold value defined hotspots more conservatively than the minimum threshold, but retained more information than the maximum threshold, and did not differ substantively from those resulting from the fluctuating yearly thresholds (Table 1).

**Table 1:** Number of grid cells (and percentage of total) designated as benthic fish biodiversity hotspots (temporal consistency ranging from 0 years hot to 8 years hot) for Coast-wide and the North biogeographic regions and for the minimum, mean, and maximum universal thresholds and annual threshold calculated for 2003–2010.

***Number of Richness Hotspots Identified***																
	**Coast-wide (46 grid cells)**								**North Region (30 grid cells)**							
**Years Hot**	**Min (32.5)**	**%**	**Mean (34.4)**	**%**	**Max (36.3)**	**%**	**Annual Threshold**	**%**	**Min (29.1)**	**%**	**Mean (31.1)**	**%**	**Max (35.3)**	**%**	**Annual Threshold**	**%**
**0**	31	67.4%	34	73.9%	43	93.5%	37	80.4%	17	56.7%	22	73.3%	28	93.3%	20	66.7%
**1**	7	15.2%	8	17.4%	1	2.2%	6	13.0%	1	3.3%	1	3.3%	2	6.7%	2	6.7%
**2**	2	4.3%	2	4.3%	2	4.3%	0	0.0%	2	6.7%	4	13.3%	0	0.0%	5	16.7%
**3**	2	4.3%	1	2.2%	0	0.0%	2	4.3%	5	16.7%	2	6.7%	0	0.0%	1	3.3%
**4**	1	2.2%	1	2.2%	0	0.0%	1	2.2%	2	6.7%	0	0.0%	0	0.0%	1	3.3%
**5**	3	6.5%	0	0.0%	0	0.0%	0	0.0%	1	3.3%	1	3.3%	0	0.0%	1	3.3%
**6**	0	0.0%	0	0.0%	0	0.0%	0	0.0%	1	3.3%	0	0.0%	0	0.0%	0	0.0%
**7**	0	0.0%	0	0.0%	0	0.0%	0	0.0%	1	3.3%	0	0.0%	0	0.0%	0	0.0%
**8**	0	0.0%	0	0.0%	0	0.0%	0	0.0%	0	0.0%	0	0.0%	0	0.0%	0	0.0%
**Total (≥1 year hot)**	15	32.6%	12	26.1%	3	6.5%	9	19.6%	13	43.3%	8	26.7%	2	6.7%	10	33.3%
***Number of H′ Hotspots Identified***																
**Years Hot**	**Min (2.36)**	**%**	**Mean (2.42)**	**%**	**Max (2.54)**	**%**	**Annual Threshold**	**%**	**Min (2.27)**	**%**	**Mean (2.37)**	**%**	**Max (2.45)**	**%**	**Annual Threshold**	**%**
**0**	22	47.8%	32	69.6%	41	89.1%	27	58.7%	9	30.0%	17	56.7%	24	80.0%	19	63.3%
**1**	16	34.8%	9	19.6%	2	4.3%	11	23.9%	9	30.0%	10	33.3%	5	16.7%	5	16.7%
**2**	3	6.5%	4	8.7%	2	4.3%	6	13.0%	5	16.7%	1	3.3%	1	3.3%	5	16.7%
**3**	3	6.5%	1	2.2%	1	2.2%	0	0.0%	4	13.3%	2	6.7%	0	0.0%	1	3.3%
**4**	1	2.2%	0	0.0%	0	0.0%	1	2.2%	2	6.7%	0	0.0%	0	0.0%	0	0.0%
**5**	1	2.2%	0	0.0%	0	0.0%	0	0.0%	0	0.0%	0	0.0%	0	0.0%	0	0.0%
**6**	0	0.0%	0	0.0%	0	0.0%	1	2.2%	1	3.3%	0	0.0%	0	0.0%	0	0.0%
**7**	0	0.0%	0	0.0%	0	0.0%	0	0.0%	0	0.0%	0	0.0%	0	0.0%	0	0.0%
**8**	0	0.0%	0	0.0%	0	0.0%	0	0.0%	0	0.0%	0	0.0%	0	0.0%	0	0.0%
**Total (≥1 year hot)**	24	52.2%	14	30.4%	5	10.9%	19	41.3%	21	70.0%	13	43.3%	6	20.0%	11	36.7%

### Biogeographic regional analysis to evaluate spatial sensitivity of CRFD method

No hotspot delineation method is without certain constraints [31]. One criticism of the CRFD approach is that it may be influenced by the spatial extent under consideration [33]. To validate that our approach was capable of detecting temporally consistent hotspots at both relatively small and large scales, we compared the sensitivity of the method at two scales within the CCLME. We re-calculated biodiversity hotspots for grid cells within the North biogeographic region (bounded by 40.8344° N and 48.3594° N), an area with relatively low temporal variability in species diversity [35]. We then compared the number and locations of hotspots detected within the North biogeographic region to those detected when considering the entire study region.

## Results

### Defining Biodiversity Hotspots

**Fig 3:**
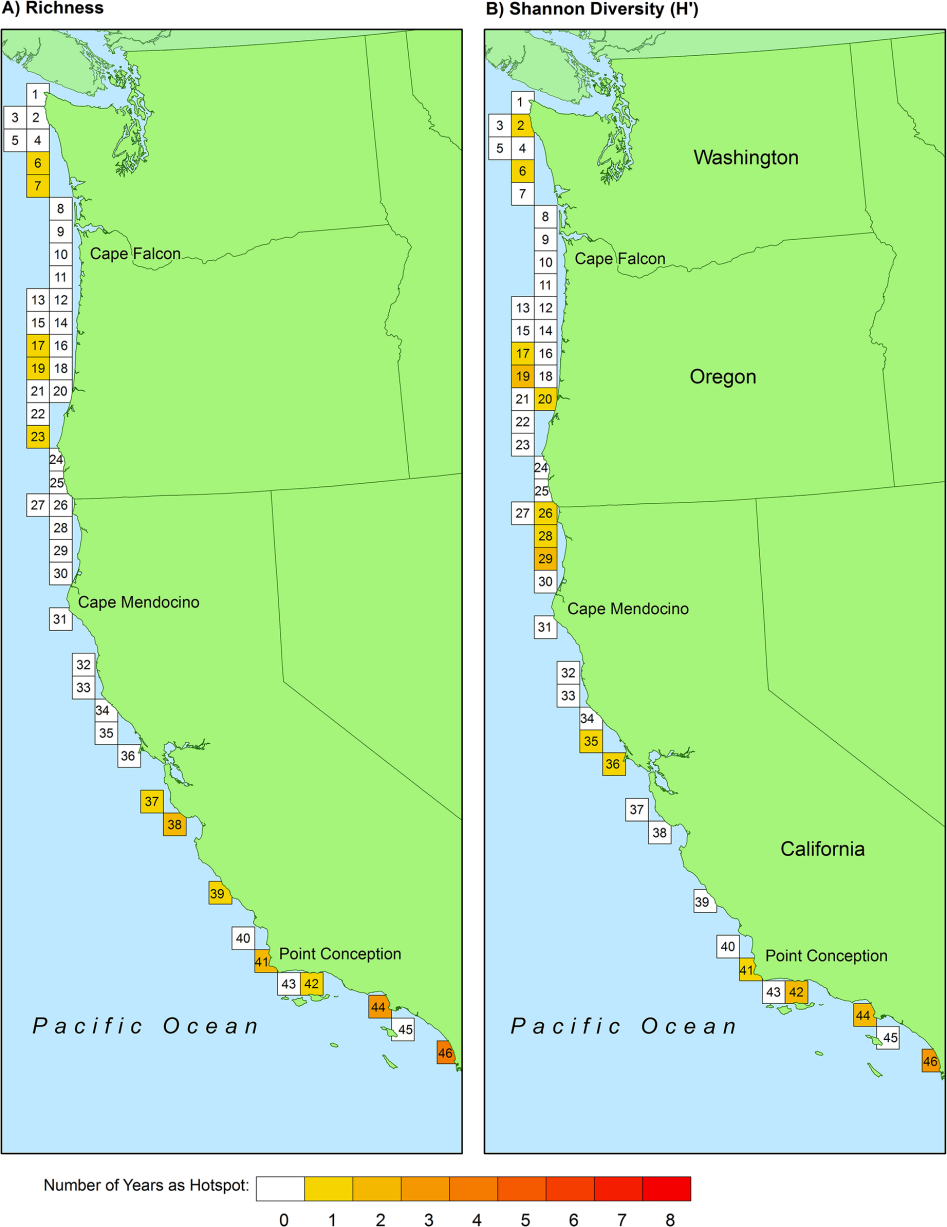
Location of 1600 km^2^ grid cells with ≥ 3 scientific trawls/year and hotspots for A) benthic fish species richness, and B) benthic fish Shannon diversity, H′. Each grid cell contains an identification number and shading indicates the number of years (out of 8, 2003–2010) that the cell exceeded the universal threshold value to be classified as a hotspot (34.4 for species richness, 2.42 for Shannon diversity H′).

**Fig 4:**
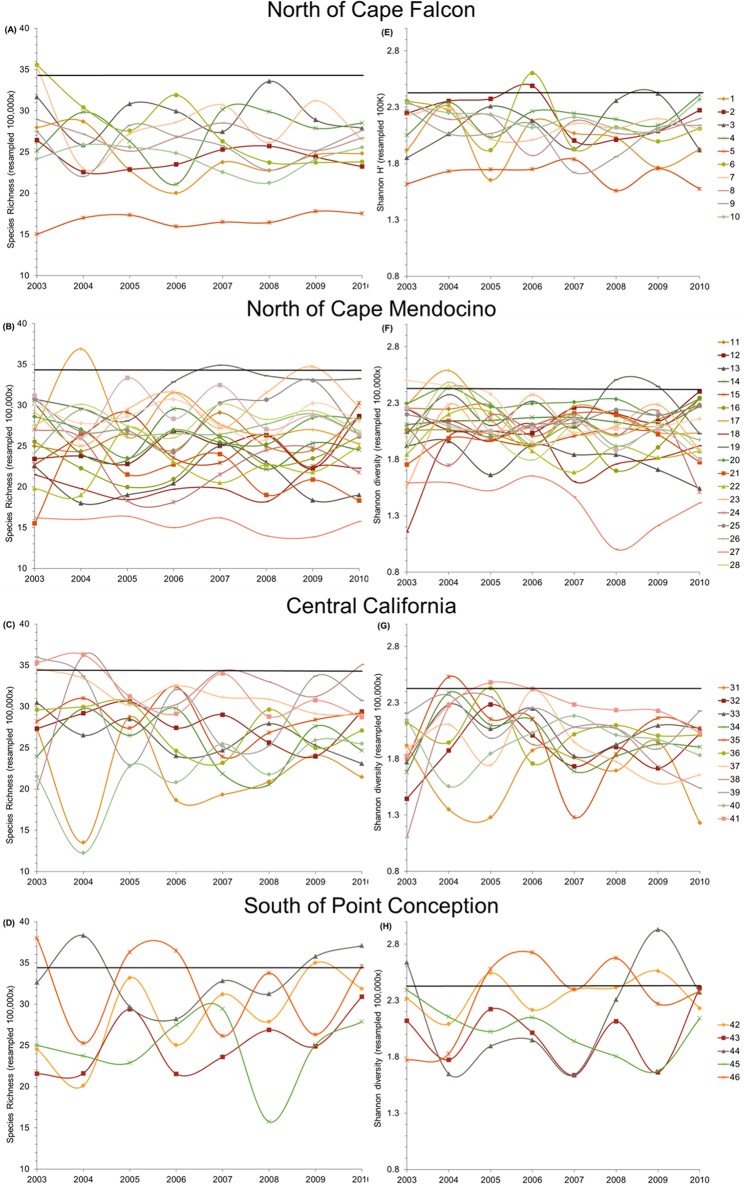
Temporal variability in benthic fish species richness (A-D) and Shannon diversity (H′) (E-H) by grid cell by region. North of Cape Falcon (A & E), Cape Falcon to Cape Mendocino (B & F), Central California (C & G), and South of Point Conception (D & H). Horizontal black lines indicate mean biodiversity threshold for hotspot designation.

Across all years, from 2003–2010, the benthic fish species richness hotspot thresholds derived from the CRFD method ranged from 32.5–36.3. Using the mean universal threshold of 34.4, 12 of the 46 total grid cells qualified as species richness hotspots at least once during the study period (Fig 3). However, only four grid cells qualified as species richness hotspots for one year or more. Only one grid cell, #46, was classified as a richness hotspot for two years consecutively, but no grid cell qualified for more than two years consecutively (Fig 3, S4 Fig). The results changed slightly when Shannon diversity, H′, was used instead of species richness (Table 1). Across all years, from 2003–2010, the H′ hotspot threshold ranged from 2.36–2.54. Using the mean universal threshold of 2.42, 14 of the 46 total grid cells qualified as H′ hotspots at least once during the study period, although only five were H′ hotspots for one year or more (Fig 3). Two grid cells, #19 and #46, were classified as H′ hotspots for two years consecutively, but no grid cell qualified for more than two years consecutively (Fig 4, S5 Fig). Only one species richness hotspot was identified from 2005–2007, and in both 2007 and 2010, no H′ hotspots were identified (Fig 4). Although fish diversity fluctuated throughout the study both spatially and temporally, many grid cells that never exceeded the hotspot threshold remained relatively stable or exhibited directional change (Fig 4).

Benthic fish hotspots were identified throughout the study region, but the most consistently hot grid cells were found south of Cape Mendocino, CA (Fig 3). The four grid cells that qualified as fish species richness hotspots for more than one year were all located along the coast of central and southern California. Three of the five grid cells that qualified as H′ hotspots for more than one year were located south of Point Conception, CA, while the other two were located between Cape Falcon, OR and Cape Mendocino, CA. The southernmost grid cell in the study area, #46, was classified as the “hottest” grid cell and qualified as a species richness hotspot for four years and a H′ hotspot for three years out of eight.

### Biogeographic regional analysis–North of Cape Mendocino

The CRFD method is sensitive to the re-sampling procedure, as re-sampling a greater number of trawls results in higher hotspot thresholds. To test the sensitivity of our approach to survey area, we examined a subset of our complete dataset. When our methods were applied to only the grid cells north of Cape Mendocino (30 grid cells), the mean universal species richness hotspot threshold decreased from 34.4 to 31.1, and the mean universal H′ hotspot threshold decreased from 2.42 to 2.37. The decline in hotspot thresholds after sub-setting to a smaller region was expected, but the percentage of grid cells identified as benthic fish hotspots remained similar in the regional analysis compared to the full analysis with respect to species richness and increased with respect to H′ (Table 1). Reduced re-sampling effort in this area reduced hotspot thresholds and the maximum biodiversity value sampled simultaneously, so lower hotspot thresholds do not always increase the proportion of hotspots identified.

**Fig 5:**
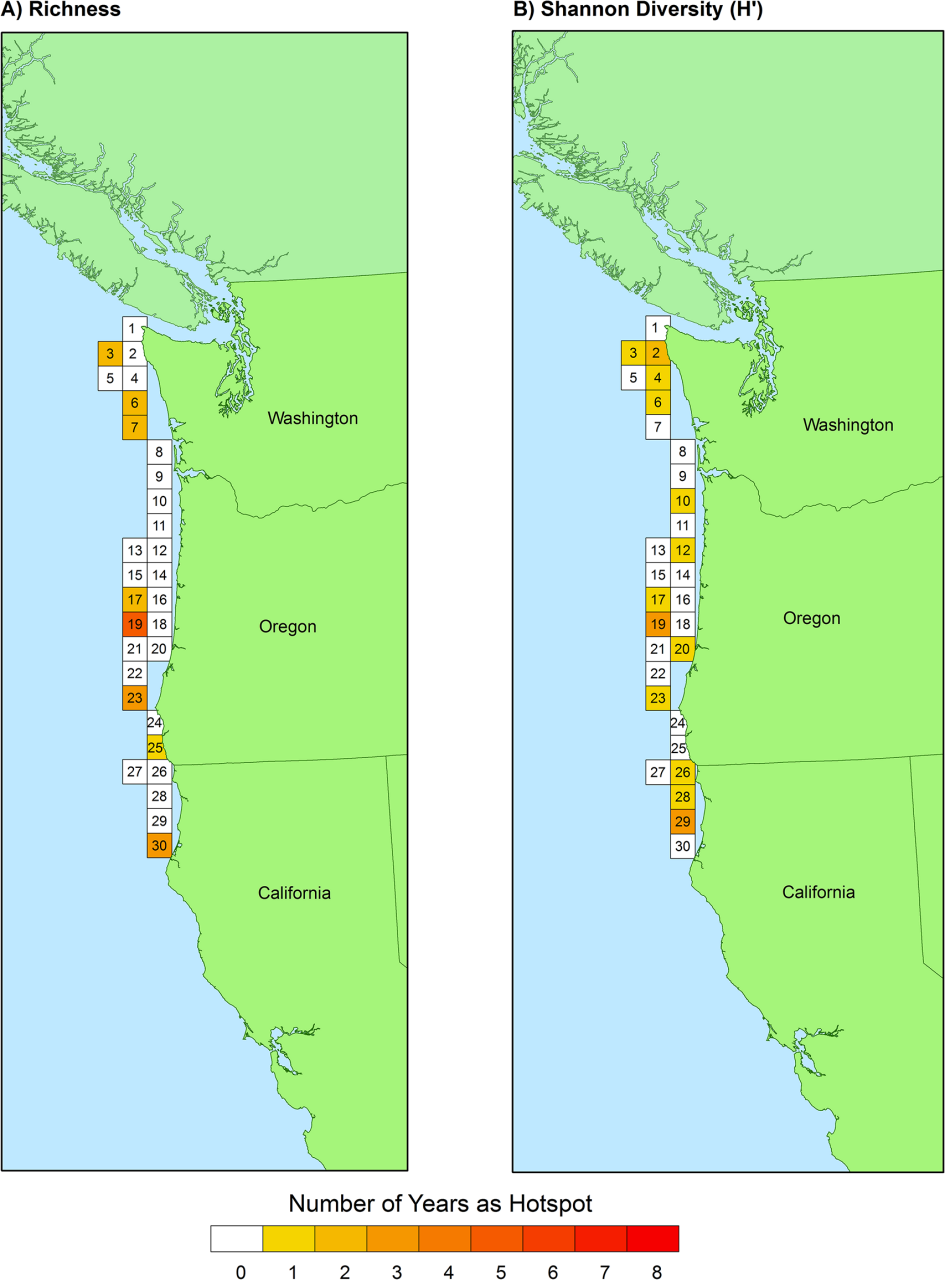
North Biogeographic region—location of 1600 km^2^ grid cells with ≥ 3 scientific trawls/year and hotspots for A) benthic fish species richness, and B) benthic fish Shannon diversity, H′. Each grid cell contains an identification number and shading indicates the number of years (out of 8, 2003–2010) that the cell exceeded the universal threshold value to be classified as a hotspot (31.1 for species richness, 2.37 for Shannon diversity H′).

Focusing our method on a smaller biogeographic region did, however, slightly increase the consistency of the fish species richness hotspots (Fig 5). More grid cells were identified as hotspots for one year or greater in the North regional analysis. In the North region, 26.7% of grid cells qualified as richness hotspots and 43.3% of grid cells qualified as H′ hotspots at least once in the eight year period, compared to the entire study extent (26.1% of richness hotspots and 30.4% of H′ hotspots; Table 1). Limiting the study extent reduced variation in biodiversity levels, as expected, and increased consistency among grid cell biodiversity measures, especially for H′. However, we still found no hotspots that were consistent throughout the entire study period.

## Discussion

We implemented an approach for identifying biodiversity hotspots in regions where species’ occurrences are spatially and temporally variable. Our objective was to determine the degree of temporal variability in biodiversity hotspots across the CCLME and contribute to the rapidly evolving concept of biodiversity hotspots. While we did identify regions of high benthic fish biodiversity, we found little consistency in hotspots over the eight year time-series, regardless of (1) which biodiversity metric was used; (2) whether thresholds were calculated per year or across all years; (3) using minimum, mean, or maximum thresholds across all years; or (4) the spatial extent for which we calculated thresholds and identified hotspots. Specifically, we found no areas containing biodiversity above our hotspot threshold through the entire study period based on the mean CRFD criteria, and no grid cell was designated as a hotspot for greater than 50% of the time-series. Moreover, in some years, no hotspots were identified (e.g., Coast-wide H′: 2007 and 2010). All grid cells exhibited fluctuations in biodiversity resulting in non-sequential hotspot classification. Altogether, these results indicate high variability in benthic fish biodiversity throughout soft sediment habitats in the NE Pacific Ocean, suggesting that a spatially explicit understanding of biodiversity in this region is insufficient for identifying conservation priority areas and that long-term data is required to appropriately identify candidate conservation areas for these taxa.

When we restricted the analysis to the North biogeographic region of the US West Coast, additional benthic fish hotspots were identified. Even though this restriction in spatial extent corresponded with lower variability in biodiversity, it failed to increase the overall temporal consistency of hotspots. This suggests that our method is not sensitive to spatial extent, at least at the scales examined here. While our method accounted for the effect of unequal sampling effort on fish diversity, it homogenized richness values among samples within grid cells and thus may have minimized the “hotspot signal” from areas of high biodiversity that were also highly sampled (such as near the Channel Islands, CA). In addition, our sampling scope was limited by trawl coverage. The 1600 km^2^ grid cells employed here are quite large and may have been too coarse to identify smaller-scale biodiversity patterns. That said, our grid cell size is not outside the range of current management areas [36]. Our work highlights the need for scientists and managers to carefully consider *a priori* the relevant scale when designing studies for measuring and monitoring biodiversity.

This inconsistency in biodiversity through time is supported by other studies and has been attributed to variability in both biotic and abiotic conditions. Toole et al. (2011) found high seasonal and inter-annual variability in species assemblages in deep water sites off the coast of central Oregon [37]. They attributed this variability to fluctuations in populations of short-lived species and differences in annual recruitment. A study of demersal fish diversity off the coast of Iceland showed a high degree of temporal variability and a northward shift in maximum species diversity over an 11-year period, which was correlated with warmer sea surface temperature [12]. Recent modeling of dispersal and retention of coral-reef organisms among Caribbean regions also found inconsistent population stability through time when populations were fragmented [38]. For the CCLME, annual variation in recruitment may drive some of the inconsistency of hotspots, as cool years associated with La Niña conditions may not support as much larval settlement and may ultimately lead to lower biodiversity in a given region, especially for short-lived species [14,16,39]. Furthermore, disturbance from fisheries, either from direct harvest, bycatch or habitat destruction, could also influence the temporal dynamics of biodiversity [40–43]. It is important to note that although we failed to detect temporal stability in hotspots derived from benthic fish diversity values, case studies for less vagile taxa may yield different results. We caution that consistent areas of high biodiversity may occur in the CCLME [44,45] and that results are taxa-specific and dependent on the temporal extent of the dataset.

While the drivers of biodiversity may differ across marine systems or taxa, understanding how ecological communities fluctuate through space and time is necessary for management and/or conservation planning. For example, Maxwell et al. (2013) created a novel metric for identifying the cumulative impacts of multiple anthropogenic stressors on marine predator distributions in the California Current System [46]. By combining this metric with tracking data on diversity of predator taxa, they determined that there was high spatial variability in species and cumulative impact distributions. Of particular interest was their finding that some of the highest impacts on marine predator distributions were localized to U.S. National Marine Sanctuaries. Thus, hotspot delineation in marine systems may not only be complicated by temporal shifts in the drivers of biodiversity, but these drivers may also be spatially complex and often synergistic. In addition, Link et al. (2013) found that variability in certain key ecosystem processes and biodiversity was greater in *a priori* identified biodiversity hotspots than coldspots, suggesting that variability in and of itself is a key attribute of biodiversity hotspots [47]. Taking together our findings and previous research, temporal variability in marine biodiversity is a key attribute to consider when identifying already spatially complex biodiversity hotspots.

To test the generality of our findings, additional long-term studies across multiple spatial scales from major biomes (e.g. terrestrial, freshwater, and other marine ecosystems) should be used. We recognize that in a system as dynamic as the California Current, an eight-year study may not be long enough to capture the full range of biodiversity variability and consequently identify temporally consistent hotspots. However, in research-limited systems such as ours it is essential to take advantage of the limited data that are available. Our approach could also be used in the future for longer studies and in other ecosystems, both marine and terrestrial. Fortunately, as in our study, some datasets already exist that can be used to answer this question of temporal consistency. The North American Breeding Bird survey, National Ecological Observatory Network (NEON), and the ICES International Bottom Trawl Survey are examples of available datasets for evaluating the temporal component of biodiversity hotspots. Researchers may also need to account for sampling design limitations when using existing datasets that were not originally designed for the desired spatial scale or the specific purpose of identifying biodiversity hotspots. For example, in our case study, we used data from the WCGBTS, which is a long-term program designed to monitor groundfish populations for fisheries management. This program sampled locations that were randomly chosen each year, rather than repeatedly sampled from fixed locations. This resulted in spatially and temporally uneven sampling effort that we addressed with our resampling procedure. Similar data aggregation and randomized resampling techniques may need to be applied to other existent datasets. Regardless, in data-poor systems it remains clear that utilizing publicly available datasets, including those generated by citizen-science programs, can yield substantial insight into long-term ecological patterns that are valuable for informing conservation and management practices.

The concept of biodiversity hotspots should be refined to encompass both spatial and temporal dynamics as ecological communities respond to a changing climate and environmental perturbations [48]. Species distributions may diverge from contemporary ranges with anthropogenic forcings, further highlighting the need to understand the dynamics of biodiversity and incorporate adaptive strategies in conservation planning [13,49,50]. Conservation tools that address spatiotemporal dynamics in biodiversity are already in use (e.g. protecting spawning/breeding grounds and wildlife movement corridors), which can be used as templates for better incorporating temporal variability. Recognition of the temporal variability in biodiversity may help to explain research findings and temper expectations regarding the success of conservation areas, such as slow recovery or unsubstantial changes in biodiversity throughout recently designated conservation areas [51,52]. Continual monitoring of biodiversity after the establishment of conservation areas is crucial for evaluating whether protection efforts remain focused within areas of high biodiversity through time and for recognizing when adaptive management strategies may be needed. Furthermore, these large-scale assessments can help to determine whether or not static, small-scale conservation areas may be insufficient to adequately protect biodiversity. Our results suggest that greater attention to temporal variability is needed in the context of biodiversity hotspots and conservation planning. Continual monitoring and adaptive management, which anticipates these spatiotemporal dynamics in biodiversity, is imperative to ensure that limited resources are applied in a well-informed, targeted manner.

## Supporting Information

S1 File Additional information on methods and data organization. (PDF)

S2 File Construction of cumulative relative frequency distribution and identification of Biodiversity Hotspot Thresholds. (PDF)

S1 Table List of families and number of species, of which were identified to the family-level or lower, included in the West Coast Groundfish Bottom Trawl Survey for 2003–2010. (PDF)

S1 Fig Species richness and Shannon diversity before (A and D) and after (B and E) resampling procedure, given the initial number of trawls per grid cell. Standard deviations of species richness and Shannon diversity (C and F, respectively) per grid cell, given the number of trawls following the resampling procedure, are also shown.
(TIF)

S2 Fig Cumulative relative frequency distribution (CRFD) curves used to generate biodiversity hotspot thresholds for fish species richness from 2003 to 2010 (A-H). Thresholds derived for each year were averaged to define a final universal threshold, which was used to determine hotspots. The dotted vertical line designates the highest point on the curve (z; *relative species richness*) that intersects with the 45° tangent line (dashed line), which we then used to calculate the corresponding threshold (*x*_*0*_).
(TIF)

S3 Fig Cumulative relative frequency distribution (CRFD) curves used to generate biodiversity hotspot thresholds for Shannon diversity H′ from 2003 to 2010 (A-H). Thresholds derived for each year were averaged to define a final universal threshold, which was used to determine hotspots. The dotted vertical line designates the highest point on the curve (z; *relative Shannon diversity H′*) that intersects with the 45 degree tangent line (dashed line), which we then used to calculate the corresponding threshold (*x*_*0*_).
(TIF)

S4 Fig Annual location of 1600 km^2^ grid cells and hotspots from 2003 to 2010 for fish species richness. Each grid cell has shading to indicate if it qualified as a hotspot in the given year (richness > 34.4).
(TIF)

S5 Fig Annual location of 1600 km^2^ grid cells and hotspots from 2003 to 2010 for fish Shannon diversity, H′. Each grid cell has shading to indicate if it qualified as a hotspot in the given year (H′ > 2.42).
(TIF)
