# Supplementary material for: Evaluating Temporal Consistency in Marine Biodiversity Hotspots
Source: PLoS One. 2015 Jul 22;10(7):e0133301. doi: 10.1371/journal.pone.0133301 (PMC4511790; doi:10.1371/journal.pone.0133301)
Supplement: S1 File — (PDF) [file pone.0133301.s001.pdf]

Data organization

Due to changes in scientific crew and survey protocols through time, there was inconsistent identification to a taxonomic level, e.g. a species might be identified to the family level or species-level within or across years. We followed these rules to remove or consolidate extraneous taxonomic classifications: 1) all eggs and larval stages were removed, 2) juveniles and adults were combined into one species, 3) if a genus was present, but no individual species from that genus, then the taxon was retained, and likewise for higher order taxonomic classifications, and 4) if a genus and a species from that genus both existed, but the total weight of a species was less than the genus, the groups were consolidated into the genus. The last step was a precaution against double counting species and genera, as it was likely there was a higher degree of uncertainty in making identifications to the species level for these taxa. In all, this process would tend to create a bias towards under-counting species. The trawl methodology employed does not provide a standardized measure of pelagic species due to the variability in the time and distance traveled through the water column; therefore, pelagic species were removed. Habitat associations were classified based on a variety of information sources (i.e. FishBase, Encyclopedia of Life, or printed literature).
