# Supplementary material for: Evaluating Temporal Consistency in Marine Biodiversity Hotspots
Source: PLoS One. 2015 Jul 22;10(7):e0133301. doi: 10.1371/journal.pone.0133301 (PMC4511790; doi:10.1371/journal.pone.0133301)
Supplement: S2 File — (PDF) [file pone.0133301.s002.pdf]

CRFD Methods

The two axes of the cumulative relative frequency distribution (CRFD) curve were defined as follows:

$$x = \frac{z}{z_m} \qquad (1)$$

where z is the biodiversity value for a grid cell and z_m is the maximum value for the study region, and

$$y = \frac{M(x)}{N} \qquad (2)$$

where $M(x)$ is the number of grid cells in which a value smaller than x occurs and N is the total number of grid cells for the region (Bartolino et al. 2010). The tangent to this curve $f(x)$ at a point x_0 has a slope of $f'(x_0)$. The hotspot threshold was selected from the highest biodiversity value corresponding to the point x_0 on the curve having a tangent with a 45° slope, or where:

$$f'(x_0) = 1 \qquad (3)$$

(Bartolino et al. 2010). At this point on the curve, the relative increase in the biodiversity variable is proportional to the frequency, offering a natural breakpoint between hotspots and non-hotspots (Bartolino et al. 2010). When more than one 45° tangent was present on the curve for a year, we derived a conservative threshold from the highest corresponding x_o.
