# Supplementary material for: Evaluating Temporal Consistency in Marine Biodiversity Hotspots
Source: PLoS One. 2015 Jul 22;10(7):e0133301. doi: 10.1371/journal.pone.0133301 (PMC4511790; doi:10.1371/journal.pone.0133301)
Supplement: S1 Table — (PDF) [file pone.0133301.s003.pdf]

Family	Number of species
Agonidae	8
Alepocephalidae	3
Ammodytidae	1
Anarhichadidae	1
Anoplopomatidae	1
Argentinidae	1
Arhynchobatidae	4
Bathymasteridae	1
Batrachoididae	1
Bythitidae	2
Caulophrynidae	1
Chimaeridae	1
Clinidae	1
Cottidae	24
Cryptacanthodidae	2
Cynoglossidae	1
Embiotocidae	10
Etmopteridae	2
Etmopteridae/Scyliorhinidae	2
Hexagrammidae	4
Hexanchidae	1
Liparidae	2
Liparidinae	13
Macrouridae	7
Merlucciidae	3
Moridae	1
Myliobatidae	1
Myxinidae	2
Nettastomatidae	2
Ophidiidae	3
Osmeridae	4
Paralichthyidae	3
Percichthyidae	1
Petromyzontidae	1
Pleuronectidae	20
Psychrolutidae	3
Rajidae	4
Rhinobatidae	1
Rhinochimaeridae	1
Salmonidae	1
Sciaenidae	2
Scorpaenidae	55
Scyliorhinidae	2

Serranidae	1
Somnosidae	1
Squalidae	1
Squatinidae	1
Stichaeidae	1
Stromateidae	1
Synodontidae	1
Triakidae	3
Trichiuridae	3
Trichodontidae	1
Triglidae	2
Uranoscopidae	1
Zoarcidae	12
**Total Species**	**233**
