# Supplementary figures and images for: Evaluating Temporal Consistency in Marine Biodiversity Hotspots
Source: PLoS One. 2015 Jul 22;10(7):e0133301. doi: 10.1371/journal.pone.0133301 (PMC4511790; doi:10.1371/journal.pone.0133301)

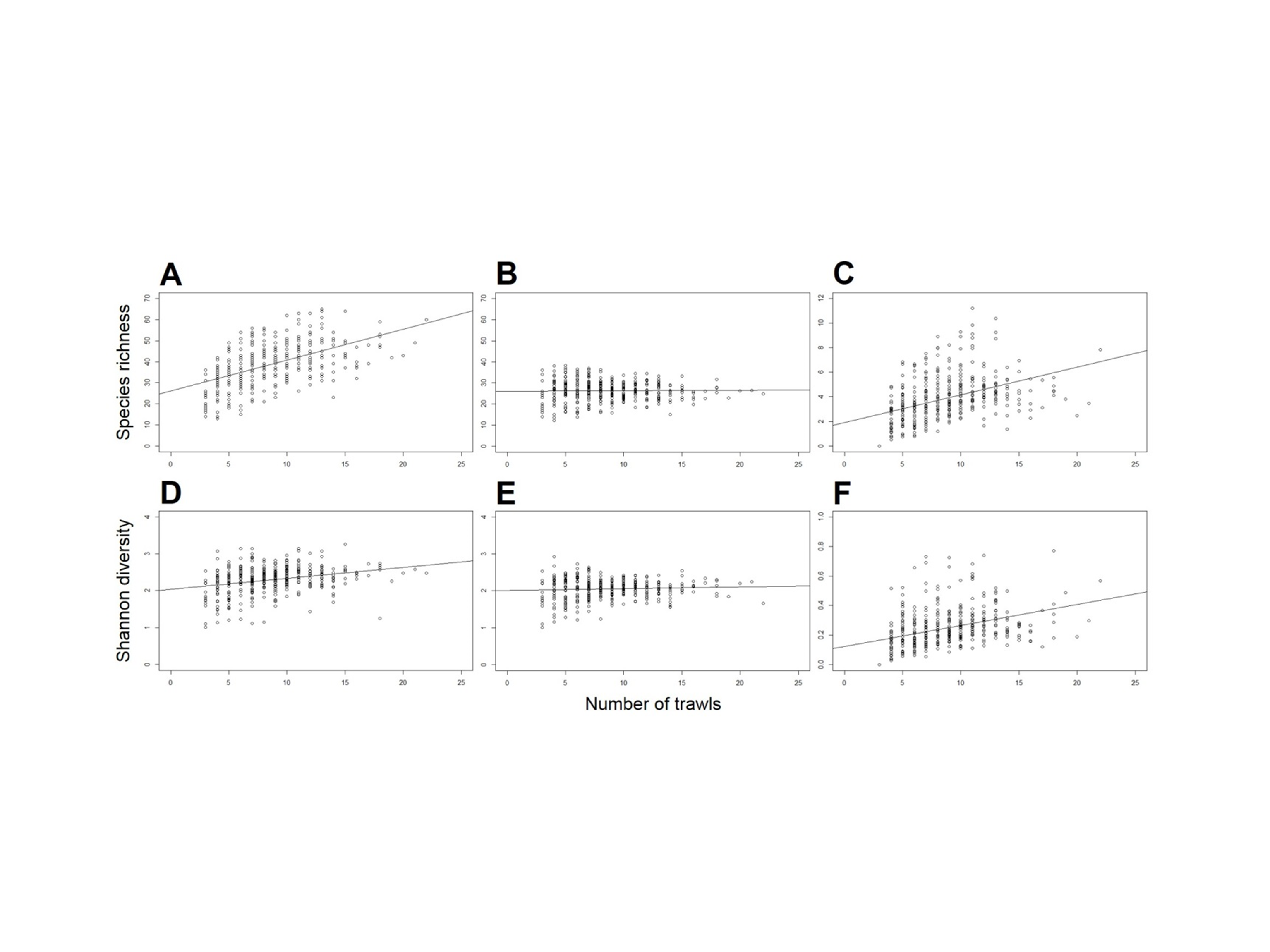

Supplement: S1 Fig — Standard deviations of species richness and Shannon diversity (C and F, respectively) per grid cell, given the number of trawls following the resampling procedure, are also shown. (TIF) [file pone.0133301.s004.TIF]

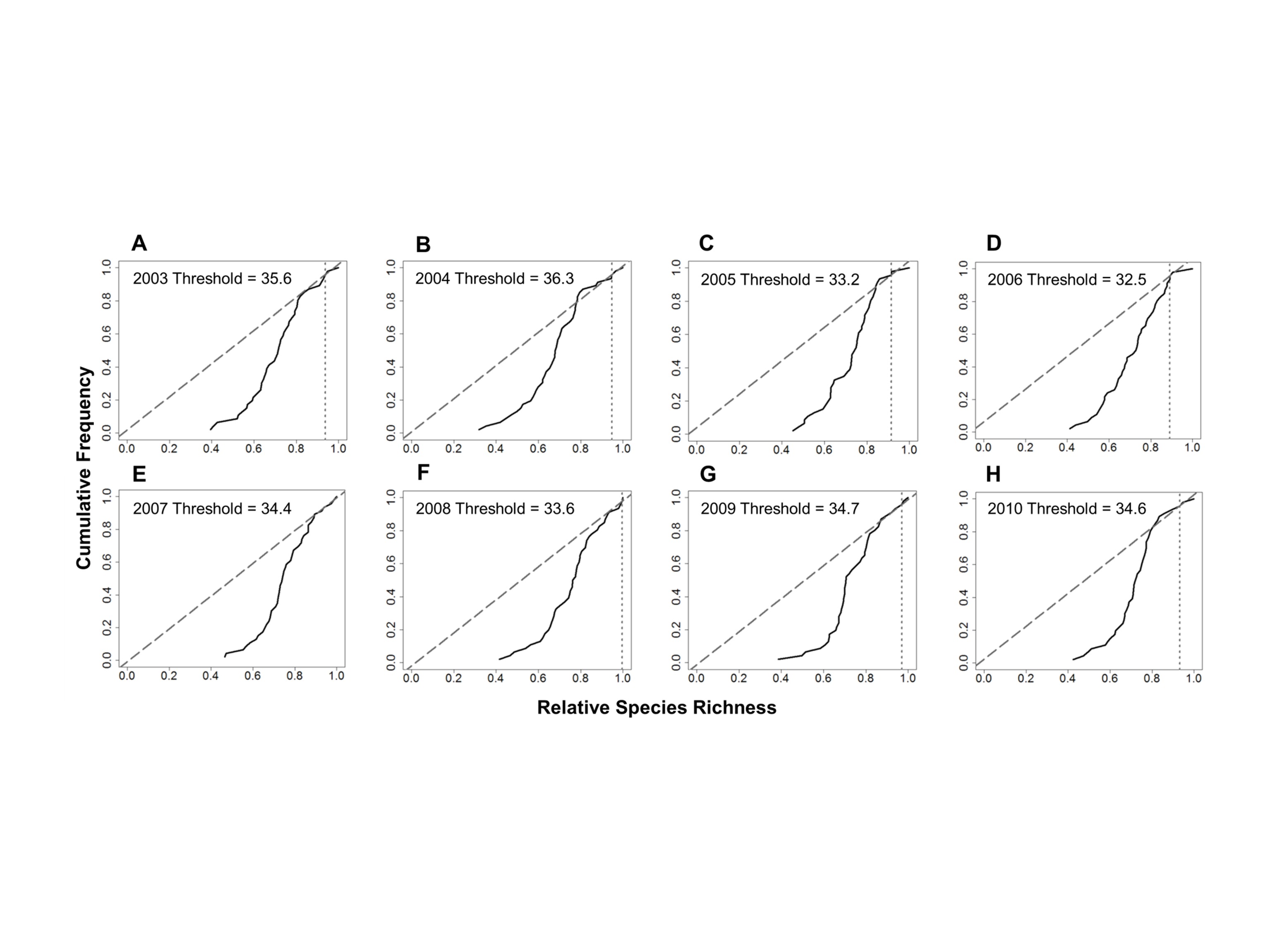

Supplement: S2 Fig — Thresholds derived for each year were averaged to define a final universal threshold, which was used to determine hotspots. The dotted vertical line designates the highest point on the curve (z; relative species richness) that intersects with the 45° tangent line (dashed line), which we then used to calculate the corresponding threshold (x 0). (TIF) [file pone.0133301.s005.TIF]

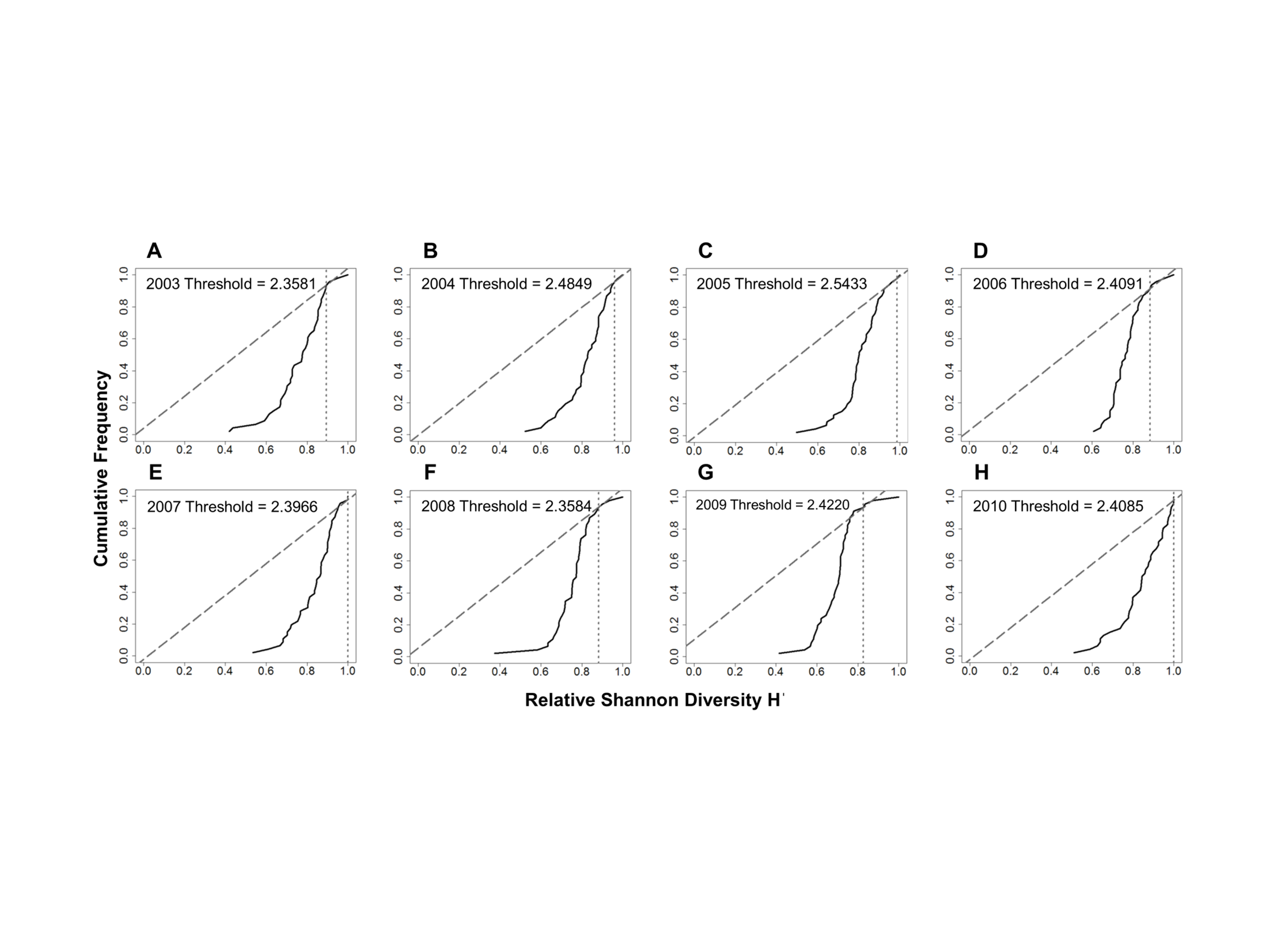

Supplement: S3 Fig — Thresholds derived for each year were averaged to define a final universal threshold, which was used to determine hotspots. The dotted vertical line designates the highest point on the curve (z; relative Shannon diversity H′) that intersects with the 45 degree tangent line (dashed line), which we then used to calculate the corresponding threshold (x 0). (TIF) [file pone.0133301.s006.TIF]

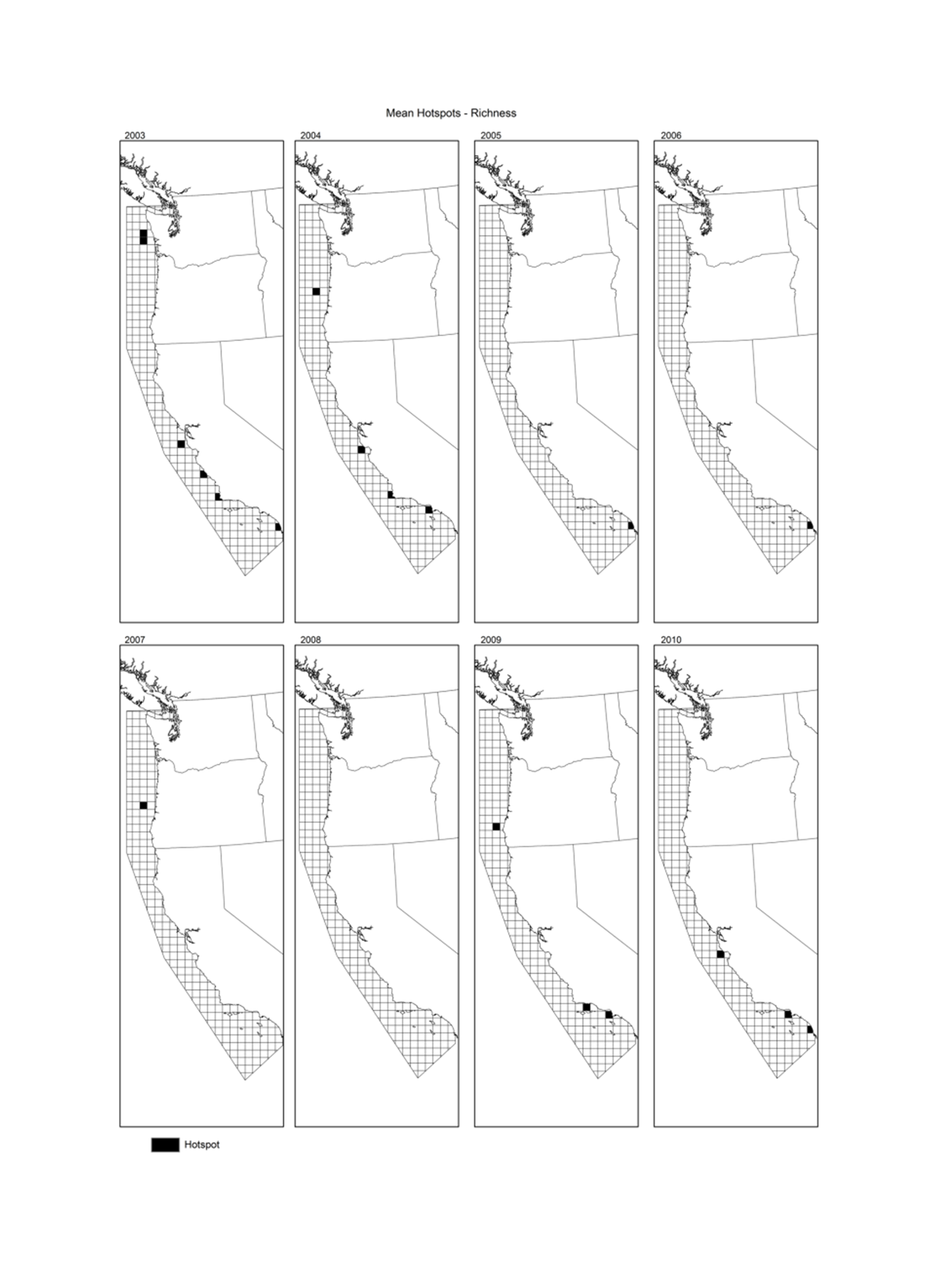

Supplement: S4 Fig — Each grid cell has shading to indicate if it qualified as a hotspot in the given year (richness > 34.4). (TIF) [file pone.0133301.s007.TIF]

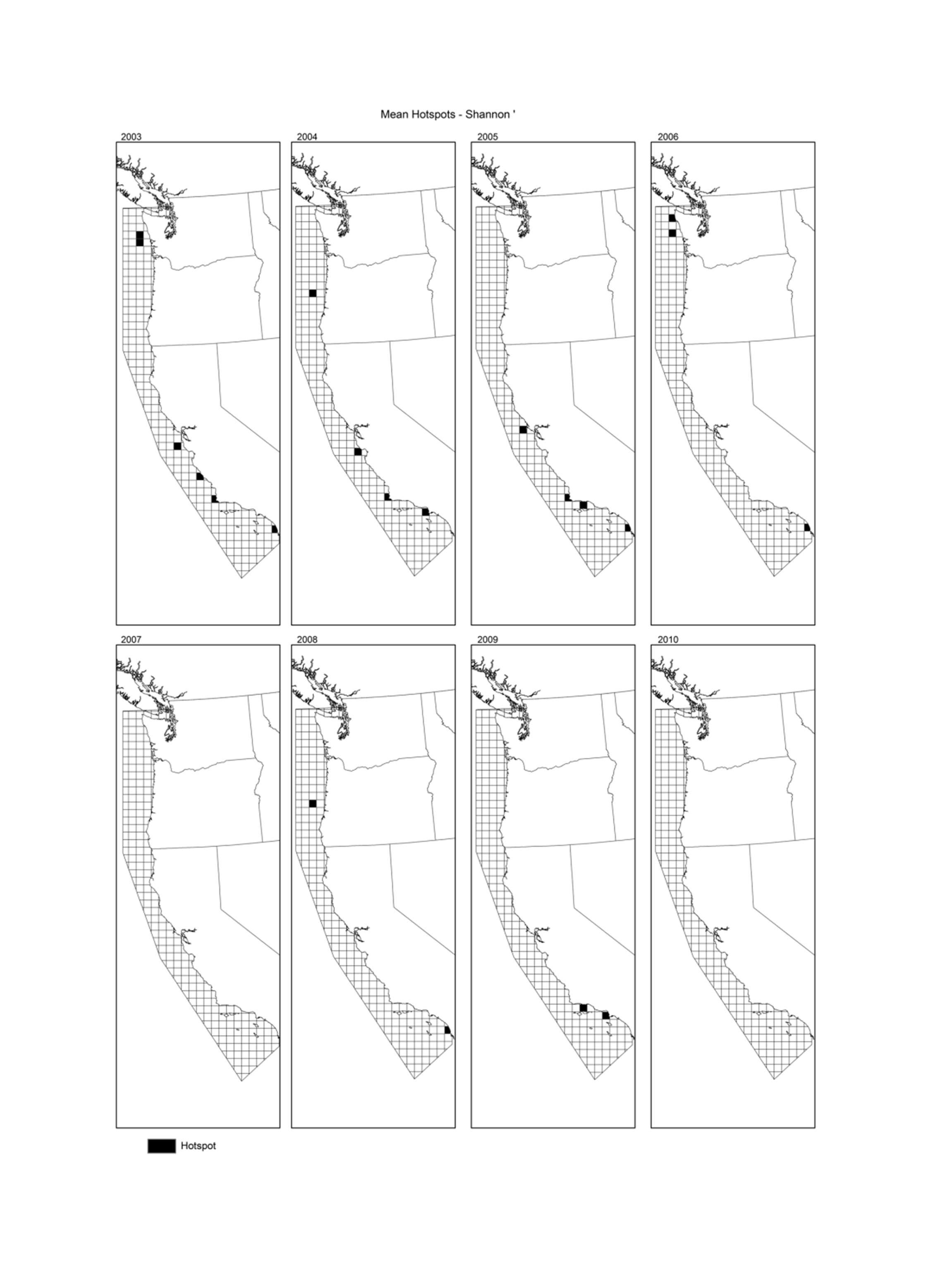

Supplement: S5 Fig — Each grid cell has shading to indicate if it qualified as a hotspot in the given year (H′ > 2.42). (TIF) [file pone.0133301.s008.TIF]
